# Targeting Glucose Metabolism in Cancer Cells as an Approach to Overcoming Drug Resistance

**DOI:** 10.3390/pharmaceutics15112610

**Published:** 2023-11-10

**Authors:** Andrea Cunha, Patrícia M. A. Silva, Bruno Sarmento, Odília Queirós

**Affiliations:** 1 UNIPRO—Oral Pathology and Rehabilitation Research Unit, University Institute of Health Sciences—CESPU (IUCS—CESPU), 4585-116 Gandra, Portugal; andrea.cunha@iucs.cespu.pt (A.C.); patricia.silva@cespu.pt (P.M.A.S.); bruno.sarmento@i3s.up.pt (B.S.); 2 1H—TOXRUN—One Health Toxicology Research Unit, University Institute of Health Sciences—CESPU (IUCS—CESPU), 3810-193 Gandra, Portugal; 3 i3S—Instituto de Investigação e Inovação em Saúde, Universidade do Porto, 4200-135 Porto, Portugal; 4 INEB—Instituto de Engenharia Biomédica, Universidade do Porto, 4200-135 Porto, Portugal

**Keywords:** tumor microenvironment, tumor metabolism, glycolysis, Warburg effect, resistance, nanoparticles

## Abstract

The “Warburg effect” consists of a metabolic shift in energy production from oxidative phosphorylation to glycolysis. The continuous activation of glycolysis in cancer cells causes rapid energy production and an increase in lactate, leading to the acidification of the tumour microenvironment, chemo- and radioresistance, as well as poor patient survival. Nevertheless, the mitochondrial metabolism can be also involved in aggressive cancer characteristics. The metabolic differences between cancer and normal tissues can be considered the Achilles heel of cancer, offering a strategy for new therapies. One of the main causes of treatment resistance consists of the increased expression of efflux pumps, and multidrug resistance (MDR) proteins, which are able to export chemotherapeutics out of the cell. Cells expressing MDR proteins require ATP to mediate the efflux of their drug substrates. Thus, inhibition of the main energy-producing pathways in cancer cells, not only induces cancer cell death per se, but also overcomes multidrug resistance. Given that most anticancer drugs do not have the ability to distinguish normal cells from cancer cells, a number of drug delivery systems have been developed. These nanodrug delivery systems provide flexible and effective methods to overcome MDR by facilitating cellular uptake, increasing drug accumulation, reducing drug efflux, improving targeted drug delivery, co-administering synergistic agents, and increasing the half-life of drugs in circulation.

## 1. Introduction

The conversion of normal cells or benign tissue into neoplastic precursors usually corresponds to malignant transformation. Additional alterations bestow these cells with unlimited proliferative potential, dissemination and metastasis, resulting in tumor progression [1]. In order to sustain the acquired features, metabolic reprogramming is essential. Changes in cellular metabolism promote the fast production of adenosine triphosphate (ATP) and an increase in the synthesis of biomolecules, including nucleotides, lipids and amino acids. Several mechanisms are known to modulate cancer metabolism, which affect essential pathways for both energy production and carbon metabolism, such as glycolysis and the tricarboxylic acid (TCA) cycle. As a consequence of these alterations, there is an increased consumption of glucose and also of glutamine in tumor cells in order to maintain their metabolic requirements [2]. Metabolic reprogramming is one of the emerging characteristics of tumor progression and is crucial to support the energy needs of cells during their continuous growth and proliferation. This metabolic reprogramming is also a key factor in the development of cancer resistance to treatment [2,3]. Often, during these treatments, cancer cells adapt, altering their metabolic pathways and becoming less susceptible to therapies. Targeting and exploiting such metabolic changes can be a promising approach to improve the chance of curing cancer. For this, the development of metabolism-targeting nanoparticles, carrying multiple therapeutic agents, are increasingly being exploited, aiming to overcome drug resistance and thus constituting an appellative tool in future cancer therapies.

## 2. Glucose Metabolism

**Figure 1:**
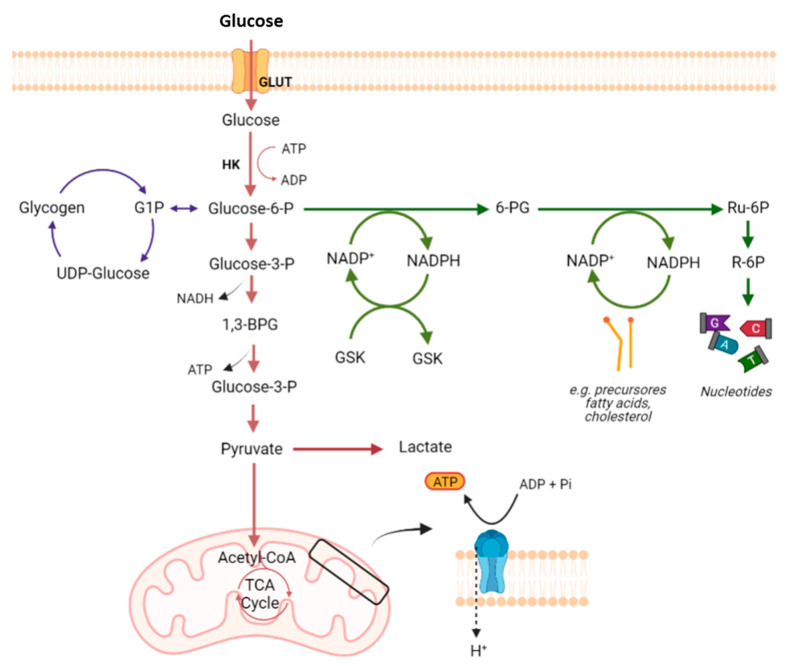
Glucose metabolism in mammalian cells. Illustrative scheme of glycolysis, TCA cycle, and the electron transport chain (red). Glucose from the blood stream is uptaken by the cells, converted into G6P by HK and posteriorly in pyruvate. In the absence of oxygen, pyruvate is converted into lactate, whereas in the presence of oxygen, the pyruvate is completely oxidized into Acetyl-CoA, which enters the mitochondrial TCA cycle. The generated NADH are then fed the OXPHOS-producing ATP (blue). The PPP (green) synthetizes the ribose-5-phosphate, which is needed for nucleic acid synthesis, and NADPH. The excess glucose is used to synthetize glycogen, via glycogenesis (purple). Created by the Authors with BioRender.com. ATP: adenosine triphosphate; G6P: glucose-6-phosphate; HK: hexokinase; NADH: nicotinamide adenine dinucleotide; NADPH: nicotinamide adenine dinucleotide phosphate; OXPHOS: oxidative phosphorylation; PPP: pentose phosphate pathway; TCA cycle: tricarboxylic acid cycle.

Most mammalian cells have glucose as their preferred metabolic substrate, which is used in the cytoplasm and/or mitochondria to provide energy for cell maintenance and proliferation [4] (Figure 1). Glycolysis, a metabolic pathway that does not require oxygen, partially oxidizes into two pyruvate molecules, producing two moles of ATP and two moles of nicotinamide adenine dinucleotide (NADH) per mole of consumed glucose [2,4]. In the presence of oxygen and active mitochondrial systems, healthy cells oxidize most of the pyruvate in the mitochondria, producing most of their ATP in this way (32 molecules of ATP from 1 single glucose molecule) [4,5]. When the anaerobic pathway is used, the pyruvate from glycolysis is reduced to lactate by the cytoplasmic enzyme lactate dehydrogenase (LDH), to regenerate the oxidized form NAD^+^ for glycolysis, producing 16 times less ATP per consumed glucose. The monocarboxylate transporters (MCTs) will then transport the excess lactate produced out of the cell through a proton-symport mechanism [4,5].

The first step in the glucose metabolism consists of its entrance into the cell. Glucose transporters (GLUTs) belong to the solute transporter (SLC2A) family of proteins and are present in many tissues/cells of the body, e.g., brain, erythrocytes, adipocytes, and liver, where they mediate glucose uptake [6]. The fourteen different isoforms of GLUTs are subdivided into three distinct protein classes, according to their sequence homology. Each GLUT isoform has a unique tissue distribution, substrate specificity, and a specific physiological function [7]. All GLUT proteins were originally assumed to catalyze the transport of hexoses into and out of cells. This is clearly the case for the class 1 GLUT proteins (GLUTs 1–4 and 14). However, class 2 (GLUTs 5, 7, 9 and 11) and class 3 (GLUTs 6, 8, 10, 12 and 13) GLUT proteins do not necessarily have a primary role in catalyzing glucose transport [8]. GLUT-1 is expressed in tissues with a high glycolytic rate, such as erythrocytes, which are responsible for glucose uptake in high-need cells [6,8]. However, this transporter also plays a central role in tumorigenesis, delivering glucose into hypoxic environments. In fact, GLUT-1, a target gene of hypoxia-inducible factor-1 (HIF-1), is highly expressed in hypoxic cancer cells, allowing for the maintenance of a high metabolic rate in these cells [6].

Although there are hundreds of types of cancer, they share some specific characteristics, namely the reprogramming of the energy metabolism. Many cancer cells predominantly rely on glycolysis, instead of oxidative phosphorylation (OXPHOS), to produce energy from glucose, even in the presence of O_2_, with this metabolic shift being known as the “Warburg effect” or “aerobic glycolysis” [9]. Although OXPHOS is downregulated, cancer cells can still obtain the required ATP for cell survival and proliferation, increasing the glycolytic flux and metabolizing glucose at high rates, with lactate production [10]. This alteration in metabolism provides a selective advantage during tumor initiation and progression, sustaining the high proliferative rate of tumor cells and promoting resistance to cells. Nevertheless, in opposition to previous beliefs, this phenotype is not due to mitochondrial dysfunction and the whole ATP factory in cancer cells is important. In fact, not all tumor cell types completely restart glycolysis for the ATP supply, and some of them may equally or even predominantly use OXPHOS [11,12]. As TCA cycle intermediates are also required for amino acids, lipid and nucleotide biosynthesis, their functioning become as important as glycolysis for tumor cell metabolism. The TCA cycle is equally important for deoxyribonucleic acid (DNA) synthesis, since the synthesis of aspartate from oxaloacetate and glutamate is critical for nucleotide synthesis [2,13]. Malate, in turn, can be used separately to produce nicotinamide adenine dinucleotide phosphate (NADPH) through a distinct pathway [2,14].

Many TCA cycle intermediates are used in biosynthetic processes; thus, a new carbon supply is required to maintain the activity of the TCA cycle. Glutaminolysis, where glutamine is used to fuel the TCA cycle, is one of the most important anaplerotic pathways in cancer [2]. In fact, glutamine deserves special attention, as it is the second most consumed metabolite by proliferating cells [2,4]. Glutamine has been shown to be essential for the synthesis of proteins, fatty acids, and nucleotides. Once inside the cell, glutaminase (GLS) converts glutamine into glutamate. Glutamate, in turn, can be converted into α-ketoglutarate, which is an intermediate of the TCA cycle. As tumor cells proliferate at higher rates, they are more glutamine-dependent than their non-tumoral counterparts [2,15]. However, a number of other metabolites have also been described to activate the TCA cycle in tumor cells [2]. For instance, in addition to being important components of membranes, fatty acids are also important energy fuels that, when degraded, provide ATP through β-oxidation [2,15]. In addition, lactate, acetate, and branched-chain amino acids (BCAAs) can also supply carbons to the TCA cycle [16,17,18]. Of these, lactate deserves particular attention. In fact, lactate produced by glycolysis (and also by glutaminolysis) in cancer cells can be taken up by neighboring cells and converted into pyruvate, entering the mitochondria and producing ATP by OXPHOS. Both efflux and uptake of lactate mainly occur via the MCT1 and MCT4, and this transport mechanism is important in tumor growth and in the inhibition of cell death mechanisms. Furthermore, it was also reported that a symbiosis between glycolytic and oxidative cells can occur, mediated by these transporters [5,19]. In fact, under anaerobic conditions, even in healthy cells, pyruvate is reduced to lactate and secreted into the extracellular space, mainly via MCT4. On the other side, lactate can be taken up by the aerobic cancer cells or by the stromal cells, mainly by MCT1 (and sometimes by MCT2), and further converted into pyruvate that can be used in oxidative conditions by these cells, sparing glucose for the more hypoxic and glycolytic cancer cells. The heterogeneity of tumors may be a possible explanation for this symbiotic model [5]. Tumors are not metabolically homogeneous and different cancer cells preferentially use particular catabolites [20]. Cancer cells are continuously adapting their metabolism, depending on the metabolism of the specific type of cancer and also on its stage, being influenced both by genetics and by the microenvironment [21]. In this way, although sometimes some cancers rely more on aerobic glycolysis and others on OXPHOS, the type of metabolism is not always cancer-specific and it is very often possible to see both types of metabolism in a heterogeneous tumor. According to this, in certain cancer types, e.g., lung cancer, both glycolytic and oxidative metabolic phenotypes were observed in different regions within the same tumor [5]. Indeed, depending on their microenvironment, tumor cells from the same tumor can be divided into subgroups: highly glycolytic with a lower OXPHOS in hypoxic conditions with defective vasculature, where nutrients and oxygen are greatly reduced, and vice versa in normoxic regions, near functional blood vessels [5]. Also, in experimental models of breast, ovarian and prostate carcinomas and sarcomas, stromal cells have been shown to produce catabolites that can be oxidatively metabolized by cancer cells, thus revealing a metabolic coupling between stromal and cancer cells [20]. In this way, and although aerobic glycolysis is a phenotype associated with cancer, OXPHOS is not only often functional, but is also important to cancer proliferation and growth, depending on the cancer type and stage. Supporting this, recent data have demonstrated that certain cancers, such as breast cancer, pancreatic ductal adenocarcinoma, melanoma, and lymphomas, rely mostly on OXPHOS [22]. Furthermore, a Gepia analysis of a five gene signatures associated with OXPHOS (ATP6V0B, ATP6V1C1, ATP6V1E1, TIMM9, and UQCRH) also showed a higher expression of these genes in these cancer types, in addition endometrium, cervical, ovarium, thymus and liver cancers (http://gepia.cancer-pku.cn/index.html (accessed on 27 October 2023)). On the other hand, and also based on Gepia, the analysis of genes associated with a glycolytic phenotype (e.g., Glut1, LDHA, HK2, MCT4) mainly showed higher expression in the lung, esophagus, head and neck, glioblastoma, kidney or colon and rectum. This has been also shown in several reports using cancer cell lines. Such differences in the energetic profile can impact the way cancer cells respond to treatment. Table 1 summarizes the energy profile of different cancer cell lines, as well as the effect of antimetabolic agents, based on this. The basal levels of monocarboxylate transporters MCT1 and MCT4 in these cell lines, and the effect of these agents on their expression, when described, is also shown. In fact, MCT1 and MCT4 are main players in the metabolic pathway preferentially used by cancer cells. WMCT4 is mainly involved in the efflux of lactic acid, and is thus more expressed in glycolytic cancer cells and often downregulated when cancer cells shift their metabolism to OXPHOS; however, many cancer cells rely on MCT1 for the uptake of lactic acid that can be used in oxidative processes [23]. For instance, the leukemic cell line NB4 presents a more glycolytic phenotype and shows a good response to the anti-glycolytic agent 2-Deoxyglucose (2DG), a nonmetabolizable glucose analogue that inhibits glycolysis, whereas the more oxidative leukemic cell line, THP1, is resistant to this agent, and sensitive to oligomycin, which targets mitochondrial respiration [24]. In these cell lines, it a higher expression of MCT1 than MCT4 was observed in the oxidative cell line THP1, while in the glycolytic cell line NB4, the expression of both transporters was found, due to the dual role that MCT1 plays in both the influx and efflux of lactic acid, in contrast to MCT4, which is mainly involved only in its efflux. A little surprisingly, in THP1 cells, an upregulation of MCT4, but not of MCT1, was observed by the oxidative substrate lactate and by VEGF [25]. Breast cancer is other kind of cancer that can present different mechanisms of energy production, depending on the cancer type. The triple-negative breast cancers usually rely on OXPHOS as the energetic metabolic pathway. According to this, it has been shown that the OXPHOS inhibitor IACS-010759 induced cell death and inhibited oxygen consumption rate in the triple-negative breast cancer cell line MDA-MB-468 [26]. In another breast cancer cell line, MCF-7, which is estrogen and progesterone receptors (ER and PR)-positive, both kinds of metabolism were found, showing the plasticity of cancer cells to adapt to their microenvironment. MCF-7 cells are sensitive to the antiglycolytic agents 2DG, 3-bromopyruvate (3BP), dichloroacetate (DCA), Iodoacetate (IAA) and lonidamine, but also to the OXPHOS uncoupler Carbonyl Cyanide m-chlorophenyl Hydrazone (CCCP). All these agents induced cell death and potentiated MCF-7 cells for treatment with the conventional anticancer drugs paclitaxel (PTX) or doxorubicin (DOX). Again, in these cell lines, in the oxidative cells, only MCT1 is observed at the plasma membrane, while in MCF-7, both transporters were found [27,28,29]. In another example of oxidative cells versus glycolytic ones, it was found that the glioma glycolytic cells U251 are sensitive to the glycolytic inhibitors DCA, 2DG, resveratrol and 2-Cyano-3-(4-hydroxyphenyl)-2-propenoic acid (CHC) (an MCT1 and MCT4 inhibitor), while the more oxidative SW1088 cells are sensitive to phenformin, in addition to DCA and 2DG [23,30,31,32,33,34]. Again, a higher expression of plasma membrane MCT4 was found in the more glycolytic cells [35,36]. Thus, in general, it was observed that more glycolytic cancer cell lines are strongly affected by glycolytic inhibitors like 3BP, 2DG, DCA, IAA and lonidamine, as well as resveratrol, which was found to inhibit this metabolic pathway, and to the MCT1/4 inhibitor CHC [23,30,31,32,33,34,35,36]. These cell lines commonly presented a higher expression of MCT4, involved in lactic acid export. The more oxidative cells are more sensitive to OXPHOS inhibitors and more resistant to antiglycolytic agents. In these cells, a lower expression of MCT4 at the plasma membrane is usually found. Concerning MCT1, the expression of this transporter was found in both glycolytic and oxidative cells, demonstrating its dual role in the uptake and efflux of lactic acid. Although some of the glycolytic inhibitors, like 3BP and DCA, are monocarboxylate analogues, and thus presumably transported by MCTs, there are few works in the literature showing their influence on MCTs’ expression and, in these cases, most of the time, no association between the treatment and the expression of the transporters was observed [27,37]. Thus, it can be assumed that their effect is usually independent of MCTs, although more studies in this area are needed. Furthermore, while some cell lines are identified as predominantly glycolytic and others as predominantly oxidative, different studies were found to attribute both of these characteristics to cell lines. These could be due to the previously mentioned fact that cancer cells present with high plasticity and can adapt their metabolism to the microenvironment characteristics, shifting from glycolysis to OXPHOS and vice-versa, what can lead to different results in the literature concerning MCTs’ expression and antimetabolic drugs’ effect. Nevertheless, some examples of cancer cell lines presenting a different effect regarding glycolytic or OXPHS inhibitors were found and are compiled in Table 1, as well as MCT1/4 expression and regulation (when the information was available), according to their energetic profile.

## 3. The Warburg Effect

In 1920, Otto Warburg postulated that cancer cells are characterized by an increased glycolytic rate, with pyruvate mostly being converted to lactate, contrary to normal cells. This phenomenon became known as aerobic glycolysis or the “Warburg effect” [2,9,57]. This observation underlies the [18F]-fluorodeoxyglucose positron emission tomography (FDG-PET) of tumors, which is used in the diagnosis of cancer and in the detection of metastasis, due to the high consumption of the glucose analogue FDG by cancer cells [58].

Originally, Warburg postulated that the increased glycolytic activity observed in cancer cells should be due to impaired mitochondrial function. In fact, mutations in TCA cycle enzymes are present in several types of cancer, such as fumarate hydratase, succinate dehydrogenase, and isocitrate dehydrogenase [9,59,60]. However, even when mitochondrial function is normal, many cancer cells still prefer glycolysis, suggesting that glycolysis is associated with advantages to these cells [9]. As several glycolytic intermediates can be used in biosynthetic pathways, it is likely that the increase in the glycolytic rate supplies the biosynthetic needs of cancer cells [61]. In fact, the high consumption of glucose allows for the energy necessary for cell growth to be obtained and, under these conditions, the PPP pathway is also favored, generating NADPH and ribose-5-phosphate, which serve as a source for the formation of new nucleotides, lipids and proteins [10,11,62]. Furthermore, the use of glycolysis may prevent the production of ROS that occurs during OXPHOS and, in this way, protects the genome of cancer cells and inhibits anoikis, allowing for the cells to survive [6,63].

The overexpression of GLUTs is essential for cancer cells to meet their high demand for glucose, which is needed for their high glycolytic rates. In addition, cancer cells often present higher levels of MCTs, since they allow for the maintenance of intracellular pH and, consequently, the glycolytic way, as they are responsible for the export of lactate. Lactate secretion may help to create an acidic extracellular tumor microenvironment (TME) that favors tumor growth, promoting migration and invasion [2,5]. The low pH found in TME activates metalloproteinases released from the cancer cells, promoting the digestion of the surrounding matrix and leading to cells’ detachment from the solid substrate [64,65]. Interestingly, cancer cells appear to be more dependent on specific isoforms of glycolytic enzymes. In fact, cancer cells may be more dependent on isoforms of hexokinase (HK2), pyruvate dehydrogenase kinase 1 (PDK1), phosphofructokinase 2 (PFK2), LDHA or pyruvate kinase isoform M2 (PKM2) [66,67,68]. In addition to these specific isoforms, to promote the glycolytic pathway, the overexpression of PDK1 inactivates the pyruvate dehydrogenase enzyme, preventing the mitochondrial conversion of pyruvate to acetyl-CoA. As a result, pyruvate remains in the cytosol and is converted to lactate [59]. The overexpression of these enzymes allows for cancer cells to easily adapt the glycolytic flux to sustain glycolytic rates and for the diversion of glycolytic intermediates to biosynthetic pathways [2]. At the same time, the excess of NADPH that is produced is closely linked to apoptosis escaping [69]. 

**Figure 2:**
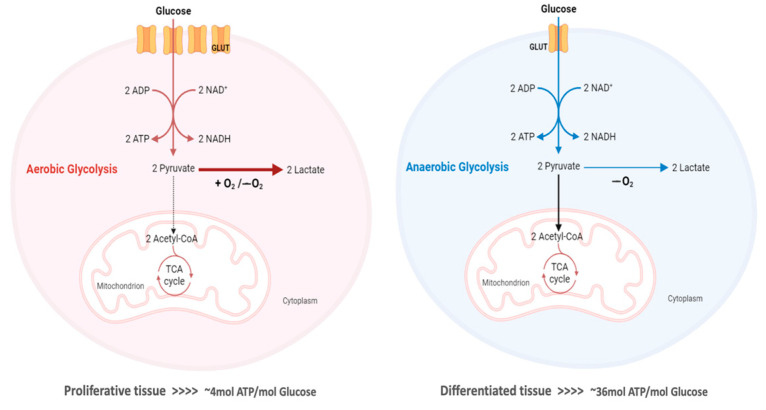
Schematic representation of the main differences between aerobic glycolysis (“Warburg effect”) in proliferative tissue and OXPHOS and anaerobic glycolysis in differentiated tissues. In the presence of O_2_, differentiated tissues (no proliferating) metabolize glucose to pyruvate via glycolysis and subsequently completely oxidize pyruvate to CO_2_ in the mitochondria (OXPHOS). At low levels of O_2_, pyruvate is partially oxidized by glycolysis, generating lactate (anaerobic glycolysis). The generation of lactate results in minimal ATP production when compared with OXPHOS. In contrast, cancer/proliferative cells predominantly produce energy through an increased rate of glycolysis, followed by a reduction of pyruvate into lactate in the cytosol, resulting in a high production of lactic acid. Created by the Authors with BioRender.com. ATP: adenosine triphosphate; OXPHOS: oxidative phosphorylation.

Thus, although ATP production through OXPHOS is more efficient, most cancer cells produce most of their ATP through glycolysis, even in the presence of oxygen [2,6,57] (Figure 2). In fact, 70–80% of human cancers present the Warburg phenotype, a metabolic alteration that results from the interaction between normoxic/hypoxic activation of the transcription factor HIF-1, oncogenes’ activation, loss of tumor suppressors, altered signaling pathways and interactions with components of the TME, as well as being associated with epigenetic mechanisms [59].

As glycolysis less efficient in energetic terms than OXPHOS, cancer cells increase their glycolytic flux by about 15 times, leading to a drastic increase in the rate of ATP production, in order to compensate the energy yield [5]. In addition, and as previously discussed, the “Warburg effect” contributes to counteracting apoptosis and promotes macromolecule biosynthesis. However, high rates of OXPHOS are displayed by some cancer cells. In fact, some cancer cells, even in a glycolytic cancer, switch their metabolism to OXPHOS, as this metabolic pathway is the predominant supplier of ATP in these cases [21,69,70]. There is a significant emphasis on enzymes like isocitrate dehydrogenase (IDH) 1 and IDH2, which catalyze the first oxidative reaction of the TCA cycle, resulting in the generation of NADH, and thus have particular importance in mitochondrial respiration [71]. For example, in some models of breast cancer, mitochondrial respiration significantly increases [72]. Thus, a “two compartment” model, also called the “reverse Warburg effect”, was proposed to reconsider a tumor metabolism where cancer cells and cells found in the TME, like cancer-associated fibroblasts (CAFs), become metabolically coupled [69,73]. In this model, cancer cells mainly use the oxidative pathway resulting from lactate obtained from aerobic glycolysis that occurs in tumor stromal fibroblasts [74]. As a result of this interaction, cancer cells induce oxidative stress by generating ROS in the form of H_2_O_2_ in CAFs, resulting in the increased production of energy-rich fuels (such as pyruvate, ketone bodies, fatty acids, and lactate) [69,75,76]. In turn, these molecules support OXPHOS in cancer cells, resulting in ATP production [69]. Even in a single tumor, OXPHOS and glycolysis contribute to different populations in different ways, since there is intratumoral heterogeneity, directing the metabolism of tumor tissue to different pathways depending on the conditions, as previously discussed [69,77]. In addition, substrates from different cancer cell populations can be shared and used, since these two different tumor cell populations may be metabolically linked [78]. For rapidly proliferating tumors, glycolysis may be more advantageous as, in addition to an abundant supply of energy, cancer cells need lipids, nucleic acids, and other glycolytic-derived intermediates for biosynthesis [69]. In fact, these types of cells need a high amount of metabolic intermediates for growth and division [79]. In differentiated tumors, which are more similar to normal tissues, with a slower growth and progression, OXPHOS may be more efficient in terms of their ATP production [69]. In addition to all the alterations inherent to glycolytic enzymes, the aberrant expression of transcription factors such as HIF-1, c-MYC and p53 also promotes metabolic reprogramming [59]. Such metabolic alterations are not only involved in cancer cells’ adaptation to hostile environments, but are also mediators of mechanisms of resistance to several conventionally used chemotherapeutic drugs, a major problem in cancer treatment effectiveness [2].

## 4. Mechanisms of Cancers’ Drug Resistance

**Figure 3:**
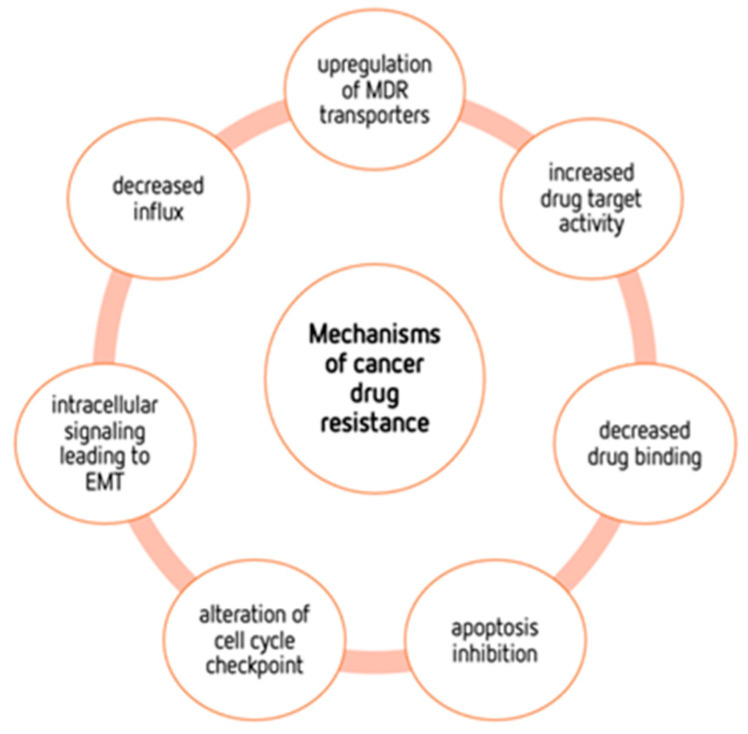
Mechanisms of chemotherapeutic drug resistance in cancer cells. This resistance may be due to intrinsic mechanisms or due to acquired mechanisms, such as the ones listed in the figure.

In the last few decades, cancer treatment has made great, promising advances. Nevertheless, despite these advances, tumors seem to always find a way to resist practically all types of anticancer therapy, hindering their treatment potential [2,80]. Cancer patients who are resistant to therapy often develop more metastases, which are the main cause of cancer-related deaths in these cases [81,82]. Thus, it is important to develop new therapeutic approaches to overcome resistance to therapy [80,81]. The growing knowledge of the molecular mechanisms of cancer has allowed for the discovery and improvement of new therapeutic compounds with a better progression-free survival. Unfortunately, this does not always translate into overall survival benefits, as resistance is one of the main problems to overcome. This resistance may be due to intrinsic mechanisms or to acquired mechanisms, which arise after the exposure of cancer cells to chemotherapeutic drugs [80] (Figure 3). 

This acquired resistance may result from several factors, namely the acquisition of mutations that cause a decrease in drug binding, an increase in drug target activity, or an upregulation of multidrug resistance (MDR) transporters [83]. For example, mutations of the TP53 gene, a tumor suppressor responsible for genome stability, are frequently observed in cancer cells and involved in cancer resistance to therapy [84]. A genomic study carried out in patients with acute myeloid leukemia demonstrated that the presence of new genetic mutations in the genes WAC, SMC3, DIS3, DDX41, and DAXX was involved in tumor resistance [85]. Another example in ovarian cancer demonstrated that the presence of secondary somatic BRCA mutations induced high resistance, especially to platinum drugs [86,87,88]. Many more examples of genetic mutations associated with cancer resistance can be found in the literature, demonstrating the complexity of the phenomenon. Other factors, such as decreased influx, intracellular signaling leading to epithelial–mesenchymal transition (EMT), alterations in cell cycle checkpoints and apoptosis inhibition are also associated with drug resistance [89]. Adaptive resistance can either be achieved through attempts to improve drug efficacy or result from the heterogeneity and adaptability of cancer cells [90]. Therefore, an important contribution to improving cancer therapy is a more complete knowledge of the resistance mechanisms, and the metabolic reprogramming can be also a major player [2,3]. This metabolic reprogramming provides a mechanism through which cancer cells can adapt and evolve to counteract the effects of therapeutic interventions. Therefore, unravelling and targeting reprogramming mechanisms is a crucial aspect of developing effective cancer treatments and overcoming resistance. 

EMT plays an important role in tumor progression, metastasis and therapy resistance and is often associated with metabolic alterations in cancer [81,91]. EMT is a highly conserved biological process that involves the transition of polarized, immobile epithelial cells into motile mesenchymal cells due to the loss of apicobasal polarity, loss of cell–cell contacts, reorganization of the actin cytoskeleton, and ability to invade the extracellular matrix as an individual cell [81]. Different studies using cancer cell lines demonstrated the responsibility of EMT in radio- or chemotherapy-driven resistance [81,92,93]. In fact, conventional anticancer drugs are mainly directed toward rapidly dividing cells, with EMT being associated with stem cell properties in cancer cells [94]. Furthermore, EMT can be involved in microenvironment modifications, causing the loss of cell–cell adherence and extracellular matrix remodeling, as well as in the interaction with the immune system, contributing to chemotherapy resistance [95,96,97]. A study demonstrated that highly proliferative non-EMT lung cells were sensitive to chemotherapy, and the emergence of EMT-derived metastases was observed after treatment [98]. Another study found that increased cellular metastasis in drug-resistant Non-Small Cell Lung Cancer (NSCLC) and, consequently, malignant progression is directly associated with the EMT phenotype [97]. EMT also promotes the heterogeneity of the tumor, and is intricately regulated by several factors, such as extracellular matrix components, diverse signal pathways, soluble growth factors or cytokines, and microRNAs [99,100,101,102]. Metabolic reprogramming is often associated with the resistance and is promoted by EMT. In fact, EMT can lead to a switch in the metabolism from OXPHOS to glycolysis, which is often associated with drug resistance [91]. Furthermore, EMT can give rise to metabolic heterogenous cancer cell populations, making treatment strategies more challenging [103].

A large number of studies on metabolism-mediated drug resistance have focused on glycolysis and the TCA cycle, including the roles of glucose and glutamine in such phenotypes [2,104,105,106]. Nevertheless, fatty acids and BCAAs may also be associated with both energy production and tumorigenesis. Concerning amino acids, their metabolism may also constitute a target for treating drug-resistant tumors. Cancer cells may be dependent on specific amino acids, like serine, glycine, proline, aspartate, and arginine. In fact, amino acid metabolism has been extensively studied and recognized as an important factor in both drug resistance and energy production. For example, Jones et al. demonstrated that increases in the level of amino acid metabolism are not related to the metabolic needs for protein synthesis, but rather to the TCA cycle, to allow for energy production. It has also been described that the overexpression of fatty acid (FA) synthase, or even the altered expression of anti-apoptotic proteins [81], induce resistance to antitumor drugs such as DOX and mitoxantrone in breast cancer cells [107].

Unfortunately, resistance to therapy not only includes resistance to conventional treatments, such as chemotherapy or radiation, but also immunological and targeted therapies [81], affecting the long-term therapeutic outcome of tumor patients [108]. Many scientific reports have shown that the MDR phenotype, which is characterized by a broad tumor’s resistance to multiple drugs and can differ either in its structure or in its mechanism of action, often correlates with the expression of active transport mechanisms responsible for the efflux of a wide variety of drugs, leading to a reduction in the effect of the drug, as there is a reduction in its intracellular levels [82,108,109]. These transporters, which are frequently highly expressed in resistant cancer cells, belong to the ATP-binding cassette (ABC) family, with P-glycoprotein (Pgp) being the first-identified and best-studied ABC transporter [108,109]. In some normal human tissues, these proteins are responsible for endogenous and exogenous substrate transport across their membranes, avoiding toxic accumulation in the organism, but in cancer they are often associated with the MDR phenotype [110]. Furthermore, several findings showed the contribution of ABC transporters to some of the remaining hallmarks of cancer [82]. 

### 4.1. ABC Transporters

**Figure 4:**
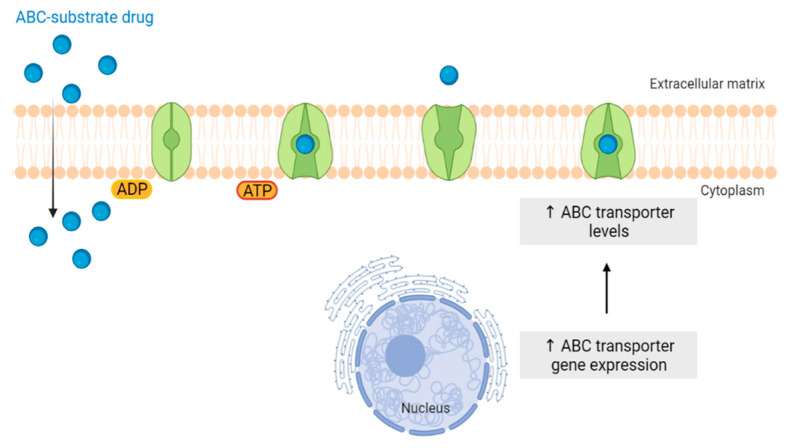
A simplified schematic diagram of ABC transporter overexpression leading to drug resistance in cancer cells. The ABC proteins (green) reduce intracellular drug concentration by actively transporting ABC substrate drugs (blue circles) out of the cancer cell, which leads to the MDR phenotype. Created by the Authors with BioRender.com. ABC: ATP-binding cassette; MDR: multidrug resistance.

The ABC transporter family is composed of seven subfamilies (ABCA to ABCG), according to their genomic sequences and the core structure of transmembrane domains, but only a few of them transport drugs; therefore, they play an important role in their bioavailability [111,112,113]. In humans, the proteins of this ABC transporter superfamily comprise at least 48 genes with diverse functions [82,114]. Given their ability to extrude several conventional antitumor drugs, recent studies in cancer research focused on the members of this superfamily to understand the reasons for the failure of chemotherapy treatment (Figure 4) [82]. 

Three major subfamilies of ABC transporters have been associated with the MDR phenotype and extensively studied: ABCB, comprising ABCB1 (Pgp/MDR1), ABCC, comprising ABCC1 (Multidrug-Resistance Protein 1 (MRP1)) and ABCG, comprising ABCG2 (Breast Cancer-Resistance Protein (BCRP)) in their respective members. These three proteins are major players in both primary and acquired resistance to chemotherapeutic drugs [82,115,116]. A key factor in the clarification of the mechanisms behind MDR was the discovery of the MDR1 and MRP1 transporters, which allowed for the identification of a variety of proteins with similar structures and transport capabilities. In addition to their role in transport of drugs, several members of the ABCB subfamily are also involved in intracellular peptides’ transport, including a key role in the presentation of major histocompatibility complex class I antigens [82]. MDR1, MRP1 and BCRP transporters can export an extensive range of chemotherapeutic compounds used in the treatment of cancer patients, making them attractive therapeutic targets [82]. In addition, cancer progression has been associated with the overexpression of some other ABC transporters, as in the case of melanoma, where a clinical correlation with ABCB5 expression was found [80,117]. To make the situation worse, several cancers overexpress more than one ABC transporter; this co-expression contributes to multiple-drug resistance [82,111]. Thus, to achieve a better clinical outcome, multi-carrier inhibitors are required [111]. For instance, the co-expression of MDR1, ABCB5 and ABCC2 was observed in a subpopulation of melanoma cells [80,117]. It has also been described that BCRP/MDR1 transporters are highly expressed in hematopoietic stem cells [80,118]. Furthermore, some studies demonstrated a possible relationship between ABC transporters and the in vivo formation of metastasis, although there is still no direct evidence of such an association [82,119].

#### 4.1.1. MDR1 Transporter

The MDR1 transporter, or Pgp, was the first drug transporter to be identified and the most pharmacologically active and clinically important efflux pump; it is widely expressed and transports a large variety of chemical substrates [113,120,121]. Variations in the efficiency of its transport may result from single-nucleotide polymorphisms in its encoding gene [111,122]. It has been reported that MDR1 expression triggers a delay in apoptosis as the response to apoptotic stimuli, both in cancer and non-cancer cells. This process was reverted when Pgp inhibitors were used [82,123,124]. Pgp is believed to be responsible for the MDR phenotype in most cancers [109,111], as it is a protein capable of actively pumping various drugs (e.g., DOX, vinblastine and PTX) out of the cell, thus reducing their cytotoxic efficacy [108]. Yin et al. found that resistance to chemotherapy in liver cancer stem cells is due to the overexpression of MDR1 (but also BRCP), leading to DOX efflux [125]. Another example of DOX resistance occurs in osteosarcoma cell lines, where the increased MDR1 expression is associated with the degree of DOX resistance [126]. In MCF-7 cells, the overexpression of MDR1 causes resistance to tamoxifen [127]. PTX, also known as taxol, is another important clinical drug for the treatment of malignant breast, prostate and NSCLC tumors [128]. The mechanism by which PTX affects malignant cell division is believed to include microtubule hyperstabilization and the inhibition of cytoskeletal restructuring. These processes are considered crucial to cell division [129]. Nevertheless, PTX is also a Pgp substrate, and resistance to this drug is often associated with treatment failure [128,129]. Despite its physiological importance in protecting cells from xenobiotics, Pgp overexpression in clinical specimens in breast, kidney and lung cancer patients led to a poor response to chemotherapy, resulting in low survival rates [111].

#### 4.1.2. MRP1 Transporter

MRP1 is a lipophilic anionic pump, which may increase resistance to antitumor drugs [130]. MRP1 has a wide variety of substrates, triggering it to confer resistance to anthracyclines, epipodophyllotoxins, vinca alkaloids and camptothecins [114]. Like other efflux pumps, MRP1 expression is associated with other processes, namely redox homeostasis, steroid, and lipid metabolism, and in the pathophysiology of different disorders [110]. It is also described that MRP1 is able to transport bioactive lipids and steroids, suggesting that the protein has additional functions during cancer growth and progression, besides the described resistance to chemotherapy drugs [131]. Although MRP1 and Pgp both belong to the ABC family of transporters, they present different levels of resistance to different families of drugs [114,132].

MRP1 overexpression is related to drug resistance in acute myeloblastic, glioma, lymphoblastic leukemia, head and neck cancer, NSCLC, neuroblastoma, melanoma, prostate, breast, kidney, and thyroid cancer [111]. In neuroblastoma, for example, MRP1 knockdown was found to reduce the mitotic index in a neuroblastoma cell line xenograft [82], and high levels of MRP1 have been used as predictors of a worse response to chemotherapy [133]. Indeed, it was discovered that the reduction in the tumor expression of MRP1 was enough to augment the antitumor effect of epirubicin in a xenograft model of NSCLC [134]. It is also important to identify specific factors that regulate ABC transporter expression in cancer contexts, specifically those of MRP1. For instance, some studies showed that p53 mutations promoted increased MRP1 expression and tumor immune-cell infiltration [135]. This correlation was verified by Zhou et al., in a study which MRP1 was correlated with the immunological infiltration of several cells of the immune system, namely B cells, CD8+ T cells, CD4+ T cells, macrophages, neutrophils and dendritic cells. The presence of these immune system cells contributes to heightened resistance to immunotherapies [136]. 

#### 4.1.3. BCRP Transporter

BCRP is involved in the efflux of exogenous and endogenous substrates and drugs, being related to several types of multidrug-resistant cancers, such as acute lymphoblastic leukemia, liver metastases, gastric carcinoma, fibrosarcoma, NSCLC, glioblastoma and myeloma [111]. A mouse model of BRCA1-associated breast cancer demonstrated that, in the group of genetically modified animals (Brca1−/−p53−/− mice), BCRP overexpression resulted in acquired resistance to topotecan treatment, whereas its knockdown improved the survival rate of these animals [137]. It was also reported that BCRP and CD133 co-expression can identify tumor-initiating cells in melanomas [80,117]. However, while the BCRP mechanisms involved in MDR are clear, clinical trials for BCRP inhibitors have provided few satisfactory results [116]. The reasons for clinical failure are diverse. One of the primary factors is associated with the restrictions on the use of BCRP function inhibitors due to their potential to elevate the plasma concentration of drugs that are substrates of BCRP. This elevation in substrate concentration, particularly for drugs with a narrow therapeutic index, can result in severe side effects. [138]. Another reason is the fact that many drugs are transported not only by BCRP but also by Pgp and other ABC transporters [139]. In addition to these limitations, it has been noted that BCRP inhibitors may exhibit toxicity. An example of this is the fungal toxin fumitremorgin C (FTC), the first BCRP inhibitor to be described, which, because of its neurotoxicity, was not suitable for use [140,141,142]. Although, to date, the most promising candidate is Ko143, this inhibitor does not have a specific effect since, at high concentrations, it also inhibits Pgp [142,143,144].

Since ABC transporters are overexpressed in several types of cancer and they are related to chemotherapy treatment’s ineffectiveness and a worse prognosis, their inhibition may be a way to prevent MDR and improve the prognosis [110]. Most inhibitors are designed to target MDR1, although there are also many cancer-related cell substrates that are exported by the ABCC subfamily [114]. However, the clinical use of ABC inhibitors was not very successful, making the discovery of a more effective strategy urgent. Moreover, when drugs are administered, they can also non-specifically target the ABC transporters of nontumor tissues, leading to side effects [111]. Furthermore, the high doses that are necessary to achieve this inhibition cause high toxicity in the brain and kidneys due to their possible accumulation [145]. The co-administration of inhibitors of these pumps and chemotherapeutic drugs can be one of the main strategies to improve the effectiveness of treatment, but more specific and precise delivery systems are still needed to avoid adverse side effects [114]. Another approach, which will be further detailed, could be the use of metabolic inhibitors, as these proteins, which are strongly associated with cancer therapy resistance, are ATP-dependent.

### 4.2. Metabolic Alterations Involved in Drug Resistance in Cancer 

Recently, it has been shown that the response to first-line chemotherapy treatment largely depends on the metabolism of cancer cells, which can be reprogrammed during the treatment [5]. The development of tumor-cell-associated resistance due to drug-induced selective pressures demonstrates specific resistant metabolic characteristics [105]. Several conventional chemotherapeutics activate apoptosis, killing cancer cells. However, if cancer cells find mechanisms to avoid chemotherapy’s cytotoxic effect, they will escape this programmed cell death and, as a consequence, the cancer will grow [106]. Several mechanisms are involved in the development of drug resistance in cancers, such as increased drug exportation, metabolic reprogramming and TME hypoxia [108,110]. The activation of different signaling pathways with the expression of signaling molecules is also involved in different mechanisms of drug resistance [108]. It is established that cells that express MDR proteins, such as Pgp or MRP, rely on ATP as their energy source to pump out drug substrates from within the cells. Consequently, the heightened expression of these proteins results in increased drug efflux due to the surplus production of cellular ATP, thereby inducing drug resistance [146]. Furthermore, as previously mentioned, TME plays an important role in the progression of cancers. Cancer cells have a greater need for nutrients to produce the necessary energy and sustain their anabolic needs. Thus, the availability of nutrients influences the proliferation rate of cancer cells. Despite this need, cancer cells have metabolic plasticity, which allows for them to adapt to conditions of reduced nutrient availability, and may, in turn, remodel the TME [147]. With changes in metabolism, the tumor microenvironment undergoes changes to ensure its survival, namely hypoxia, acidosis, and the formation of stroma cells. These changes, besides being particularly adverse to normal cells, are involved in the development of chemoresistance. Hypoxia can be caused by increased oxygen consumption, the rapid growth and proliferation of the tumor and also by the lack of a vascular system in certain tumor zones [10,147]. On the other hand, and as previously mentioned, hypoxia can lead to the greater use of glycolysis for the production of ATP in cancer cells, and this mechanism of obtaining energy leads to an accumulation of lactate in cells, facilitating the evasion of the immune system [148,149]. Lactate is transported to the outside of cells through the increased activity of pH regulators like ATPases, carbonic anhydrases and MCTs in order to maintain the intracellular acid-base balance. A study by Tavares-Valente shows that the inhibition of the pH regulator with concanamycin-A, cariporide, acetazolamide and cyano-4-hydroxycinnamate decreased the aggressiveness of the MDA-MB-231 cell line, a breast cancer line. A synergistic inhibitory effect was also verified in this study when these pH inhibitors were combined with DOX regarding the viability of the breast cancer cell line. These results support the interruption of proton dynamics as an antitumor strategy for breast cancer and the use of regular pH inhibitors to increase the activity of conventional therapy [149]. 

**Figure 5:**
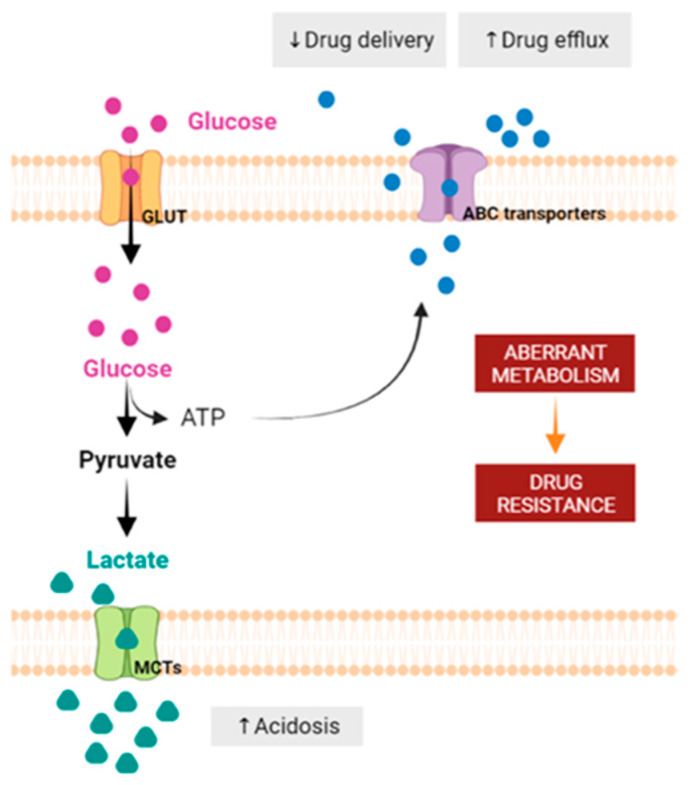
Metabolic alterations underlying the development of cancer cell drug resistance. Glycolysis upregulation is associated with ATP production and ABC transporter activity, leading to the reduced sensitivity of cells to chemotherapeutic agents. In addition, the low pH of TME, promoted by lactate accumulation and transported out of the cell by MCTs, reduces the therapeutic agent. Created by the Authors with BioRender.com. ABC: ATP-binding cassette; ATP: adenosine triphosphate; MCTs: monocarboxylate transporters; TME: tumor microenvironment.

Several studies have shown that the specific therapeutic pressure induced by drugs and the adverse conditions found in the tumor environment, namely acidity and hypoxia, lead to treatment resistance, and such resistance is also promoted by a metabolic reprogramming [110,150]. Glycolysis upregulation is one of the major metabolic modifications and is associated with ABC transporter activity, reducing the sensitivity of cells to chemotherapeutic agents [110]. Pgp activity also depends on TME characteristics and it has been shown that its activity was doubly increased in prostate cancer cells exposed to acidic media (pH 6.6) [112]. This augmentation of activity leads to an increase in the efflux of Pgp substrates, such as PTX, and thus a decrease in drug cellular sensitivity. Furthermore, the acidification of the extracellular medium reduces the uptake of several therapeutic agents, such as DOX or PTX, thus contributing to drug inaction [110] (Figure 5).

At the mitochondrial level, mitochondrial DNA (mtDNA) depletion is related to tumor progression and metastasis, and may further act as a “progression signal” for chemoresistance [106,114]. Li et al. showed that mtDNA-depleted androgen-independent prostate carcinoma cells, despite growing slowly, are highly carcinogenic, revealing an overexpression of BCRP and extremely aggressive and radio- and chemoresistant characteristics [151]. In addition, the fact that these cancer cells present a slow growth may be an advantage in their resistance to chemotherapy treatments, since the cytotoxic agents used in conventional chemotherapy have a more direct impact on rapidly proliferative cells [106,116]. mtDNA depletion in hepatocarcinoma cells resulted in cisplatin, DOX, and SN-38 chemoresistance linked with the upregulation of the MDR1 gene and MRP1 and MRP2, which are particularly involved in MDR. In colon cancer cells that are mtDNA-depleted, the upregulation of MDR1 has also been observed [117,118].

### 4.3. Metabolic Modulation as an Approach to Overcome Drug Resistance

**Figure 6:**
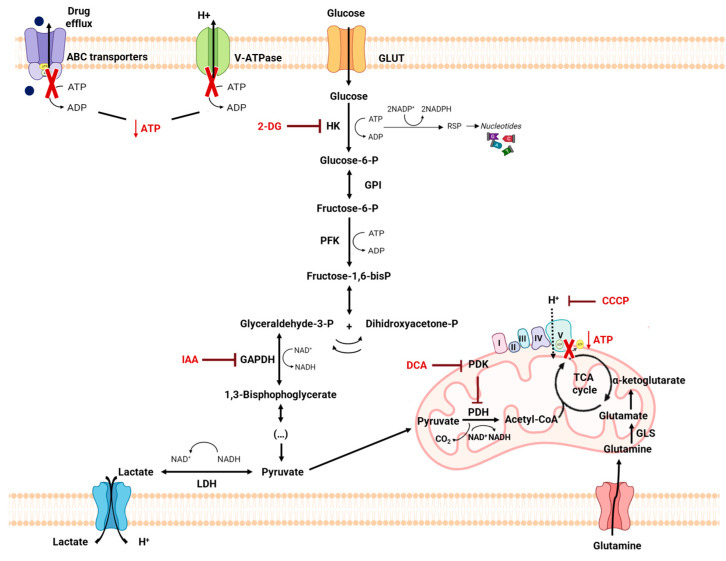
Metabolic modulation as an approach to overcome drug resistance. Glucose and glutamine metabolism, in tumor cells, supply vital components for the high requirements of both glycolysis and OXPHOS. The different compounds (IAA and 2DG) are glycolytic inhibitors. DCA inhibits PDK, reactivating PDH, and switching the metabolism from glycolysis towards OXPHOS. CCP is an uncoupler that inhibits ATP synthesis. The depletion of cancer cell energy probably leads to the inactivation of the pumps’ ABC transporters. Created by the Authors with BioRender.com. 2DG: 2-deoxyglucose; ABC: ATP-binding transporter; ATP: adenosine triphosphate; CCP: Carbonyl Cyanide m-chlorophenyl Hydrazone; DCA: dichloroacetate; IAA: iodoacetate; OXPHOS: oxidative phosphorylation; PDH: pyruvate dehydrogenase.

The metabolic reprogramming of cancer cells, besides its role in cancer proliferation and invasion, is also implicated in the acquisition of resistance to therapy in cancer patients. In this way, the recent increase in the knowledge of tumor cell metabolism and the subsequent exploration of metabolic alterations in these cells may offer an opportunity to discover new potential targets for therapeutic intervention and to overcome such resistance. This is particularly important in the different types of cancers that show resistance to drugs, to improve treatments and avoid adverse side effects. Disruption of the Warburg effect is the most often used means of sensitizing the cells to conventional antitumor drugs, exploiting cancer metabolic reprogramming [152]. Thus, glycolytic inhibitors can be used as a therapeutic strategy as they drastically decrease cellular ATP levels, which is necessary to maintain the activity of the drug efflux pumps [111] (Figure 6). This could be an effective strategy, as one of the best-described mechanisms of drug resistance is due to the increased level and/or activity of the efflux pumps that remove drugs from cells [110]. As previously described, the Warburg effect plays a significant role in therapy resistance mechanisms by contributing to metabolic reprogramming. Therefore, the use of glycolytic inhibitors alongside conventional chemotherapy may enhance the effectiveness of standard drugs by modulating metabolism. Some trials were carried out with the aim of targeting drugs in order to modulate the Warburg effect. AR-C155858, which targets MCTs 1 and 2, and AZD3965, an MCT1-specific inhibitor that partially inhibits MCT2, developed by the pharmaceutical company AstraZeneca, can have anti-cancer effects [153,154]. In a breast cancer cell xenograft model, AR-C155858 showed no significant effects on tumor growth [155]. AZD3965 has been demonstrated to inhibit the growth of several tumor cell lines, notably lymphoma [156,157]. A clinical trial in Phase I (NCT01791595) demonstrated that AZD3965 is tolerated at doses that allow for interaction with the target in advanced cancer [158]. A study demonstrated that phenformin and IAA induced a diminution in cancer cell proliferation and, when combined with conventional antitumor drugs, an increase in drug cytotoxicity was found [32]. Other drugs, such as 2DG, 3BP, DCA, lonidamine, resveratrol and apigenin, are known as inhibitors of glycolytic enzyme [159,160]. However, on the Clinical Trial website (https://clinicaltrials.gov (accessed on 20 October 2023)), most of them are under consideration in the preclinical phase. 

Amino acid metabolism can be also related to MDR phenotype, as it provides cancer cells with specific adaptive characteristics to neutralize the mechanism of action of the antitumor drugs to which they are exposed [161]. In fact, amino acids play an important role both in most biosynthetic pathways, which are upregulated in cancer cells, and in maintaining the redox homeostasis balance [113]. Among these, glutamine plays a crucial role in cancer metabolism and in drug resistance in cancer cells, since glutaminolysis supports the biosynthesis of many essential molecules [105,162] (Figure 6). The importance of glutamine is also due to the fact that it is the amino acid with the largest carbon source for the TCA cycle. In the context of tumor cells, glutamine metabolism can provide essential building blocks for the excessive demands of both glycolysis and OXPHOS [163]. In addition to its role as an essential intermediary metabolite, glutamine regulates cell survival and proliferation via signal transduction pathways, specifically the mammalian target of the rapamycin (mTOR) pathway [164], as well as the extracellular signal-regulated protein kinase (ERK) signaling pathway [165]. Additionally, this metabolic pathway may induce resistance in tumor cells against chemotherapy drugs by perturbing the delicate balance of sugar, lipid, and protein metabolism [163]. Thus, the specific inhibition of enzymes involved in the cancer amino acid metabolism may emerge as a successful therapy strategy [161]. Figure 6 shows the use of metabolic modulation with different compounds as an approach to overcome drug resistance.

For example, melanoma cells lacking argininosuccinate synthetase activity, and thus presenting with auxotrophy to arginine, were not able to proliferate under arginine deficiency in in vitro models [166]. In another example, the glutamine transporter SLC1A5 and the enzyme GLS were considerably upregulated in aromatase inhibitor (AI)-resistant breast cancer cells, and the inhibition of these proteins decreased cell proliferation [167]. PKM2 is another essential enzyme of the glycolysis pathway, an isoform that is a potential target in the search for anti-cancer drugs [153]. PKM2 is significantly upregulated in hepatocellular carcinoma, where it is associated with poor prognosis [66,168]. PKM2 inhibitors have been explored in cancer research because they have the potential to disrupt the abnormal metabolic processes in cancer cells, potentially slowing down their growth or even causing cell death. In other study, PKM2 knockdown inhibited hepatocellular carcinoma cell proliferation, migration, and invasion in vitro, as well as tumor growth in vivo. Also, in human melanoma cells, it was demonstrated that benserazide, an inhibitor of PKM2, in addition to being an aromatic L-amino acid used for the treatment of Parkinson’s disease, inhibited cell proliferation [169]. Benserazide binds directly to the PKM2, blocking its activity, and thus leading to the inhibition of aerobic glycolysis and restoration of OXPHOS [170]. Hence, blocking the primary energy production pathways in cancer cells could lead to reduced drug efflux by depleting cellular ATP, potentially reducing drug resistance [37]. Various types of cancer, when treated with a variety of drugs, present a correlation between ABC transporters and resistance phenomena, since cells expressing MDR proteins such as Pgp require ATP to be used as an energy source to pump drug substrates. Thus, drug sensitivity in cancer cells can be re-established through glycolysis and/or OXPHOS inhibition, as this inhibition will lead to ATP depletion, with a negative and specific impact on ABC transporter activity. Nakano et al. demonstrated that the suppression of glycolysis by the glycolytic inhibitor 3BP preferentially occurs in cancer cells, causing an inhibition of ATP synthesis and, consequently, of the activity of the ABC transporter. In contrast to specific inhibitors targeting a single efflux pump, this ATP depletion simultaneously inactivates all ABC transporters expressed in cancer cells, preventing the efflux of antitumor drugs and potentiating their cytotoxic effect on the cell [171]. Resistant cell lines are often chosen to study the role of the glycolysis inhibition effect in drug resistance due to the aberrant ABC transporters’ expression that expels drugs [159]. Ma et al. proposed that 2DG reversed the resistance of MCF-7 cells with an MDR phenotype and increased DOX-induced apoptosis by interfering with glucose metabolism. The process was related to the intracellular ATP depletion and, consequently, to drug efflux pump inactivation [172]. In cancer cell lines of multiple myeloma, and in leukemic cells, when treated with mitoxantrone and 3BP, a greater uptake of the chemotherapeutic agent mitoxantrone was verified. This suggested that the inhibition of glycolysis with 3BP simultaneously led to the inactivation of all types of ABC transporters in these cells, as these transporters were dependent on the ATP formed during increased glycolysis [37,159]. Other studies suggested that metformin and phenformin, antidiabetic drugs that also interfere with energetic metabolism in cancer cells, show promise in decreasing resistance through the inhibition of ABC transporters in breast cancer [32,80,173].

Metabolic adaptations in chemoresistant cells have a complex pattern involving further alterations in the reprogrammed metabolism, characteristic of cancer cells. Such modifications are mainly associated with the Warburg effect, but other players are also involved, such as amino acid and lipid metabolism, the redox state of the cell, mitochondrial reprogramming, or polyamine synthesis [3]. A profound knowledge of chemoresistant cells metabolomics is thus essential to identify metabolic targets that can be manipulated to circumvent such resistance. 

### 4.4. Self-Delivery of Nanomedicine to Overcome Drug Resistance

**Figure 7:**
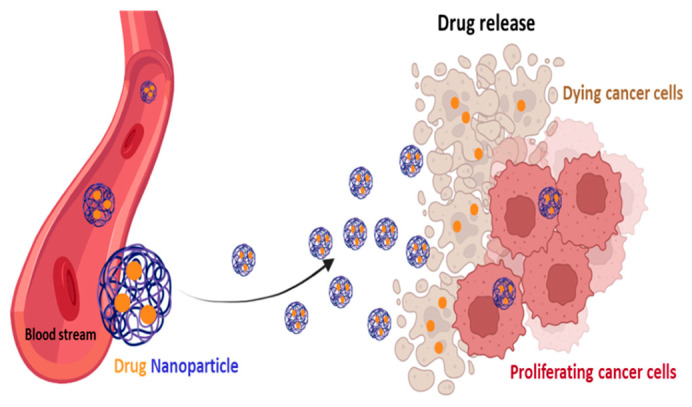
Schematic representation of nanoparticles as a drug delivery vehicle into cancer cells. The drug can be dissolved, entrapped, encapsulated, or attached to a nanoparticle matrix in order to promote therapeutic absorption, particularly in oncology. Once inside the cell, the nanoparticle is degraded through intracellular signals in order to release the drug. Created by the Authors with BioRender.com.

Chemotherapy, radiation therapy and resection surgery remain the three “gold standard” anticancer therapies [174]. Whether radiotherapy and surgery can be indicated for localized cancers, chemotherapy is considered the most appropriate treatment for most patients with metastasis and advanced cancer, as chemotherapy drugs can be widely distributed in the organism through the bloodstream [175]. Nevertheless, the development of drug resistance and the low hydrosolubility of drugs are significant problems that restrict the clinical use of currently available chemotherapy drugs [175]. Major chemotherapeutic agents include compounds like platinum complexes, DOX, vinca alkaloids, and taxanes, and primarily affect nucleic acids and protein synthesis, interfering with cell cycle and triggering apoptosis [176,177]. However, most of the standard agents approved for clinical use do not have the capacity to differentiate normal cells from cancer cells. This leads to serious side effects, especially in rapidly growing cells, as these drugs generally compromise mitosis. These cells include hair follicles, bone marrow cells and the gastrointestinal system, leading to hair loss, immune system failure, and infections, respectively [178]. Thus, the decrease in the toxicity and side effects of the main chemotherapeutic agents is an urgent problem that needs to be overcome [176]. To overcome this problem, various compounds, such as 3BP, DCA and 2DG, that interfere with metabolism, have been tested and demonstrated their ability to decrease tumor cell metabolism [37]. However, there are disadvantages to a metabolism-based approach in cancer therapy, since the metabolic pathways required for cell survival are also present in normal cells. Thus, metabolism-based treatment can face the major hurdle of non-specific toxicity [5]. To decrease their toxic side effects and increase antitumor efficacy, a number of drug delivery systems have been developed, such as albumin-bound PTX (Abraxane^®^) or liposome-entrapped PTX and DOX, which have received clinical approval, as these formulations presented enhanced security but maintained their effectiveness [175,176,179]. Several countries, namely the EU, US and Japan, approved the use of Abraxane^®^ combined with carboplatin as a first-line treatment in advanced NSCLC patients for whom curative surgery and/or radiation therapy was not an option [180]. Further investigations into the treatment of other solid tumors based on Abraxane^®^ are ongoing. The use of Doxil^®^ (liposomal DOX), with an improved safety profile in comparison to free DOX, has also been approved for clinical use in patients with multiple myeloma (NCT00103506) [179]. In other example, the combination of radiotherapy and Caelyx^®^, a pegylated liposomal DOX, led to a significant increase in the intratumoral concentration of DOX in the brain tissue of patients with glioblastoma [181]. These nanodrug delivery systems facilitate the drugs’ entry into cancer cells and reduce their export, thus promoting intracellular drug accumulation and improving targeted drug delivery. In addition to this, they allow for the co-administration of synergistic agents, and increase the half-life of drug in circulation [181,182]. In fact, in a therapeutic context, the correct combination of drugs with different mechanisms of action is needed. As the doses and efficiency of these drugs are often limited due to their toxicity, is important to develop cancer-specific delivery systems, namely drug encapsulation in nanoparticles. These systems are able to transport both hydrophobic and hydrophilic drugs, ensuring the sustained release of the drug and increasing the half-life of the drug in the bloodstream. The half-life of temozolomide, for example, was increased to 13.4 h, compared to 1.8 h for the free drug, through encapsulation in chitosan-based nanoparticles (NPs) [181] (Figure 7). The hypoxic, hypoglycemic, and acidic conditions, characteristic of the TME, are important to trigger drug release, allowing for researchers to create a TME-responsive delivery system. Furthermore, the overexpression of surface receptors by cancer cells can be used to target these delivery systems toward cancer cells through antibodies with the aim of reducing side effects in normal tissues [183].

Nanotechnology-based cancer therapies aim to find new therapeutic methodologies correlated with disease mechanisms. The use of nanoparticles to encapsulate the drugs may increase the specificity of delivery to cancer cells and decrease the interaction with other non-cancer cells involved in tumor growth and spreading [174]. Poly(lactic-*co*-glycolic acid) (PLGA), a synthetic thermoplastic aliphatic biodegradable and biocompatible polyester, is widely studied and is one of the most characterized polymers [184]. PLGA is degraded in non-toxic products (H_2_O and CO_2_) that are easily excreted [176,184]. Its polymeric NPs are degraded in vivo into lactate and glycolate. D-lactate is not metabolized prior to excretion and L-lactate is transformed into CO_2_, which is eliminated by pulmonary excretion, or converted to pyruvate, which fuels the TCA cycle. Glycolate can be directly excreted by the kidneys or can be oxidized to glyoxylate, which is, in turn, further metabolized producing glycine, serine, and pyruvate. Subsequently, pyruvate can re-enter the TCA cycle and follow the OXPHOS pathway [184]. The lactic acid (LA)/glycolic acid (GA) proportion is a good indicator not only when adjusting the degradation time, but also of the drug release rate [184,185]. Due to the absence of lateral methyl groups in GA, it has a higher hydrophilia, and thus, when higher amounts of GA are present, a higher degradation rate is observed [184,186]. Wu et al. showed that higher degradation rates of PLGA-based scaffolding were achieved when an LA:GA ratio of 75:25 was used, relative to a ratio of 85:15 [187]. Therefore, these polymeric features, as well as their size, prove to be important in adjusting the hydrophobicity, drug loading effectiveness, and pharmacokinetic profile of PLGA formulations [184,185]. The shape of the PLGA NPS appears to be another important feature, as it affects the outcome of cancer treatment. Needle-shaped PLGA NPs appear to cross endothelial cell membranes more efficiently compared to spherical forms [184]. In fact, needle-shaped PLGA NPs have been reported to significantly increase cytotoxicity. After being endocyted, these particles enter lysosomes, where they can activate apoptosis and induce cell death [184,188].

Some PLGA polymers are FDA-approved materials and various PLGA NPs formulations have been clinically introduced, such as a formulation targeting advanced prostate cancer, ELIGARD^®^ [178]. PLGA NPs were also shown to be effective in increasing the accumulation of docetaxel in gastric tumors, thus causing an increase in anticancer activity [189]. Importantly, PLGA NPs are versatile systems as they can deliver hydrophobic or hydrophilic drugs [178]. Surface adjustment with, for example, PEGylation (PEG) increases the formulation’s hydrophilicity, producing a particle with an improved blood circulation time and pharmacokinetics, preventing opsonization and absorption by the mononuclear phagocytic system [184].

Ongoing research underscores the significance of the TME in driving tumor proliferation, invasion, metastasis, and resistance to therapeutic interventions. As mentioned, the TME provides protection for cancer cells, enabling them to evade conventional treatments like surgery, radiotherapy, and chemotherapy. Furthermore, the constituents of the TME play a pivotal role in fostering therapy resistance in solid tumors. Consequently, directing interventions toward the TME presents a promising avenue for advancing the field of cancer nanomedicine. The combination of antitumor drugs with drugs that interfere with resistance mechanisms has largely been made possible by advancements in nanotechnology [190]. Hence, directing efforts toward the TME presents an innovative approach to advancing the field of cancer nanomedicine [37,191]. Nanoparticles developed in response to TME cues, such as a low pH, redox conditions, and hypoxia, enhance the pharmacokinetics and therapeutic effectiveness of nanomedicine, but also have glycolytic inhibitors [37,192,193,194]. Although not directly associated with this, and as has been shown for DCA in a lung cancer cell model, the use of nanoparticles improves the delivery of the compound, which can be important in cases of resistance. In fact, Cunha et al., with the aim of enhancing the cellular internalization of DCA, a glycolytic inhibitor, through lung cancer cells, and thereby increasing its anticancer activity, successfully achieved nanoencapsulation in PLGA [37]. In other study, the authors successfully encapsulated a glycolytic inhibitor, 2DG, in PLGA nanoparticles, and administered it to liver tumors in mice [193]. In addition to PLGA particles, 2DG was also encapsulated in liposome particles with the aim of achieving a synergistic effect with DOX. In vivo results show that this nanosystem has effective therapeutic characteristics, as well as reduced side effects [194]. Since cancer cells do not exclusively rely on ATP production through glycolysis, nanosystems loaded with mitochondrial inhibitors have also been developed. A nanoparticle designed to deplete copper specifically within the mitochondria (known as a mitochondria-targeted copper-depleting nanoparticle or CDN) was evaluated for its effectiveness against triple-negative breast cancer. The study revealed that CDNs effectively reduce oxygen consumption and OXPHOS, inducing a metabolic shift toward glycolysis and diminishing ATP production in these cells. This energy deficiency, coupled with compromised mitochondrial membrane potential and increased oxidative stress, ultimately leads to apoptosis [195]. On the other hand, some studies obtain a single nanoparticle, supplied with a glycolytic inhibitor and a mitochondrial inhibitor, with the aim of synergistically blocking both forms of energy production, such as the nanolipossoma [196,197]. In order to increase its effectiveness, NPs can be also coated with specific ligands directly targeted to cells in the TME, which promote tumor progression and aggressiveness [198,199]. Table 2 summarizes the metabolic-reprogramming-targeted, nanotechnology-based interference strategies for overcoming chemotherapy resistance.

Using NPs to direct therapy to energy metabolism and the TME could be a promising approach to sensitizing cells to conventional chemotherapy. Although the use of nanotechnology is still a recent field in cancer therapy, there is already enough evidence of its potential for successful treatment, allowing for a more accurate and specific delivery of antitumor drugs into cancer cells and avoiding many adverse side effects. Many barriers still need to be overcome regarding the success of NPs in clinical trials. Some of these barriers include the size and timing of certain NP therapies. The majority of experimental tests of NPs are cell-based and use animal models, which may not lead to convincing results in human testing. Furthermore, as the presence of metastases is a significant property of cancer, more studies should be carried out with models of cancer metastasis [201].

## 5. Conclusions

Although conventional chemotherapy is particularly toxic to tumor cells, it is often non-specific, and is responsible for the significant side effects associated with cancer treatment. However, there are differences between cancer cells and healthy cells that can be explored to increase treatment specificity against cancer. One of these differences consists of the “Warburg effect”, currently considered an emergent cancer hallmark, whereby the upregulation of the glycolytic rate in tumor cells is a key player in acid-resistant phenotypes through their adaptation to hypoxia and acidosis, as well as in tumor aggressiveness [2,9,159]. High glycolytic rates are widely reported to promote the chemoresistance of tumor cells to conventional therapy [2]. In fact, increased acidification of the extracellular space leads to lower drug stability and, consequently, lower drug efficacy. In parallel, the increased production of glycolytic intermediates promotes cell proliferation, since these are biosynthetic precursors, whereas ATP production sustains the activity of proteins involved in both drug efflux and cell division. Together, these effects underly multidrug resistance. Nevertheless, many cancer cells adapt to changes in TME, exhibiting metabolic plasticity and switching their metabolism from glycolysis to OXPHOS, and vice-versa. For example, OXPHOS could be the predominant metabolic pathway used by cancer stem cells, and is often involved in cancer resistance, metastasis, and tumor relapse [202]. Exploring specific characteristics of cancer cells, such as this change in metabolism, could be a promising strategy for the use of more effective and more specific drugs that primarily target cancer cells. In fact, metabolic changes in cancer cells can reveal specific vulnerabilities that could be targeted with precision therapies. However, the metabolic plasticity and interchange of glycolytic and oxidative cells, although occurring many times in the same cancer and being responsible for tumor heterogeneity, is not taken into account in cancer therapies. Thus, more integrated research is needed, investigating the main metabolic pathways used in different conditions and stages of each cancer type, and the influence of the TME characteristics (e.g., oxygen, pH, nutrients availability, immune components) on such metabolic adaptation and heterogeneity. An understanding of these metabolic switches, the identification of metabolic targets, and the use of combined therapies in a more targeted way through the use of nanoparticles could have a huge impact not only on the development of new drugs, but also on the ability to overcome drug resistance, one of the major problems that occurs during cancer treatment. 

This review focuses on this integrated knowledge, with a triangle of three vertices, corresponding to metabolic reprogramming and plasticity, drug-resistance mechanisms, and drug-delivery systems, serving as a promising and hopeful strategy to effectively combat cancer. 

## Figures and Tables

**Table 1:** Energy profile of different cancer cell lines, as well as the effect of antimetabolic agents and expression and regulation of MCTs based on this. Glycolytic Inhibitors—2-Cyano-3-(4-hydroxyphenyl)-2-propenoic acid (CHC), 2 Deoxyglucose (2DG), 3-bromopyruvate (3BP), Dichloroacetate (DCA) Iodoacetate (IAA), Lonidamine, Quercetin, Resveratrol, OXPHOS Inhibitors—Atovaquone, Uncoupler Carbonyl Cyanide m-chlorophenyl Hydrazone (CCCP), IACS-010759, Metformin, Olygomycin, Phenphormin.

Energetic Profile	Type of Cancer/ Cancer Cell Line	Antimetabolic Drug Effect	Expression and Regulation of MCTs	References
Mainly OXPHOS	Breast (MDA-MB-468)	IACS-010759 induced cell death and inhibited oxygen consumption rate	MCT1 expression at the plasma membrane. MCT4 is expressed on cytoplasm	[26,28]
	Cervical (HeLa)	Metformin and Rotenone promoted anoikis	MCT1 expression > MCT4 expression Hypoxia induced the expression of MCT4	[31,38,39]
	Cervical (siHa)	Rotenone decreased cell migration	2DG and rotenone increased the expression of MCT1 and CD147	[40,41]
	Leukemia (THP-1)	Resistant to 2DG and sensitive to oligomycin	MCT4 expression Lactate and VEGF increased the expression of MCT4, but not of MCT1	[24,25]
	Lung (A549)	Resistant to 3BP, DCA and 2DG	No changes were observed in MCT1 and MCT4 upon treatment with 3BP, DCA and 2DG	[37]
	Melanoma (B16F10)	Metformin and Rotenone promoted anoikis	No data	[31]
	Ovarian (OVCAR-3)	Atovaquone slowed ovarian cancer growth	No data	[42]
Mainly Glycolytic	Bladder (5637)	Sensitive to 2DG. 2DG depleted cellular ATP and potentiated the toxicity of conventional drugs	High expression of MCT1, MCT4 and CD147 Knockdown of MCT4 inhibited 5637 cancer cell line proliferation and clonogenic activity	[43]
	Colon (SW480)	Sensitive to 3BP, 2DG and DCA	High expression of MCT1, MCT2 and MCT4 3BP decreased the expression of MCT1and MCT4, but not of MCT2	[35,44,45,46,47]
	Glioma (U251)	Sensitive to DCA, 2DG, resveratrol and CHC	High plasma-membrane expression of GLUT1, MCT1, CD147 Silencing of MCT1 decreased the glycolytic phenotype	[23,30,32,33,34,48]
	Leukemia (NB4)	Sensitive to 2DG and 3BP	High expression of MCT1 and MCT4	[24,49,50]
	Lung (NCI-H460)	Sensitive to 3BP, 2DG and DCA	No association was observed between MCT1 and MCT4 expression and treatment effect with 3BP, DCA and 2DG	[37]
	Melanoma (A375)	Sensitive to 3BP	High expression of MCT1	[51]
Both glycolytic and OXPHOS	Breast (MCF-7)	2DG, IAA, DCA and CCP and 3BP induced cell death Pre-treatment with 2DG, IAA, DCA and CCCP enhanced PTX and DOX toxicity Lonidamine potentiated the effect of PTX	High plasma-membrane expression of MCT1 and MCT4. 3BP did not alter the expression	[27,29,31,52,53]
	Glioma (SW1088)	Metformin and Rotenone promoted anoikis DCA, 2DG and phenformin led to a decrease in ATP content Resistent to CHC	Low plasma-membrane expression of MCT1, MCT4 and CD147	[23,32,36]
	Liver (HepG2)	2DG, 3BP and DCA induced cell death and potentiated the effect of DOX Phenphormin inhibited proliferation	High expression of MCT1 and MCT4 and lower expression of MCT2	[54,55,56]

**Table 2:** The metabolic-reprogramming-targeted, nanotechnology-based strategies.

Metabolism Pathway	Nanoparticle	Advantages	Disadvantages	Future Perspectives	References
Mitochondrial respiration	DCA NP PLGA	Control drug delivery system of small drug molecules	Increased DCA in normal cells could lead to serious side effects	Functionalize NPs to specific tissue receptors	[37]
	CDN polymersome NPs	Induce a metabolic shift toward glycolysis Low toxicity of CDNs in healthy mice	Not applicable to glycolytic cells	Apply to other types of cancer	[195]
Aerobic glycolysis	2DG-NPs-PLGA	Control drug-delivery system of small drug molecules	Extremely low loading rate of 2DG into the 2DG-PLGA-NPs	Combination therapy with 2DG-PLGA-NPs and other therapeutic agents	[193]
	Nanoenabled Energy Interrupter	Sensitive to an acidic TME	Preferential inhibition of NPs on melanoma cells	Increase specificity for other tumor types	[200]
Aerobic glycolysis and Mitochondrial respiration	Liposome NPs	Acidic TME favorable for the decomposition of NPs	No data	Combination therapy with nanolipossoma and antitumor agents	[196]

## Data Availability

Not applicable.
